# Regarding: nailfold capillary abnormalities as indicators of diabetic nephropathy progression: a cross-sectional study in type 2 diabetes

**DOI:** 10.1080/07853890.2025.2534524

**Published:** 2025-07-15

**Authors:** Jingyuan Ma, Wenxing Fan

**Affiliations:** a Department of Nephrology, First Affiliated Hospital of Kunming Medical University, Kunming, Yunnan Province, China; b Yunnan Key Laboratory of Organ Transplantation, First Affiliated Hospital of Kunming Medical University, Kunming, Yunnan Province, China

To the Editor

We read with great interest the recent article titled “Nailfold capillary abnormalities as indicators of diabetic nephropathy progression: a cross-sectional study in type 2 diabetes” published in *Annals of Medicine* [1]. The authors should be commended for providing a comprehensive analysis of nailfold videocapillaroscopy (NVC) as a non-invasive biomarker for diabetic nephropathy (DN) progression. The innovative integration of KDIGO staging with capillary morphological data, along with the development of a semi-quantitative NVC scoring system, highlights the potential of this technique for early microvascular assessment and personalized risk stratification in type 2 diabetes.

Despite its strengths, we believe that several important limitations deserve attention to strengthen future investigations in this area. First, although the study controlled for some metabolic parameters, it did not account for non-renal microvascular complications (such as diabetic retinopathy [2] or neuropathy [3]) that may independently alter NVC results. Given the shared pathophysiological basis across these complications, future studies should include stratified analyses or multivariate adjustments to isolate DN-specific microvascular changes.

Second, the reproducibility and objectivity of NVC assessments remain a concern. While the authors performed all examinations by a single trained operator, the lack of inter-rater validation or AI-based image quantification may limit the generalizability of findings. We suggest future work incorporate blinded dual-assessor validation or explore machine learning tools for automated capillary analysis to standardize interpretation and facilitate clinical translation. Third, the study did not evaluate the incremental predictive value of NVC beyond conventional DN markers such as eGFR and albuminuria [4]. Incorporating ROC analysis, net reclassification improvement (NRI), or decision curve analysis could demonstrate whether NVC adds clinical utility in risk prediction models and helps guide decision-making in real-world settings.

In the context of the global rise of chronic diseases like diabetes, it is critical that national public health systems integrate predictive tools like NVC within a tiered emergency management framework and broader preventive health networks. The inclusion of NVC screening into community health check-ups could align with primary-level risk identification strategies, especially in resource-limited settings. Moreover, health policy should incentivize multidisciplinary models where endocrinologists, nephrologists, and community-based chronic disease managers collaborate using tools like NVC for early-stage intervention.

In conclusion, this article provides valuable insights into the microvascular pathophysiology of DN and supports the potential of NVC as a novel screening and monitoring modality. Our comments aim to enhance the translational impact of this promising technique. We look forward to future research validating its predictive value longitudinally and integrating it into comprehensive chronic disease management systems.

## Data Availability

Data sharing is not applicable to this article as no data were created or analysed in this study.
